# Place de l'hémodialyse dans la prise en charge de l'intoxication aiguë au lithium

**DOI:** 10.11604/pamj.2016.24.27.8820

**Published:** 2016-05-09

**Authors:** Jaouad El Maghraoui, Nadia Kabbali, Mohamed Arrayhani, Tarik Sqalli Houssaini

**Affiliations:** 1 Service de Néphrologie, CHU Hassan II, Equipe de Recherche REIN, Faculté de Médecine et de Pharmacie, Fès, Maroc

**Keywords:** Psychose maniac-dépressive, intoxication au lithium, trouble de conscience, hémodialyse, Manic-depressive psychosis, lithium intoxication, disorder of consciousness, hemodialysis

## Abstract

Nous rapportons le cas d'un patient âgé de 47 ans, suivi depuis vingt ans pour une psychose maniaco-dépressive sous lithium admis dans un tableau de trouble de conscience après une tentative de suicide au lithium (30 comprimés de Téralithe^®^ LP 400, forme galénique retard correspondant à 12 g de carbonate de lithium), bien amélioré cliniquement après trois séances d'hémodialyse. Cette observation illustre l'intérêt thérapeutique de l'hémodialyse au cours des intoxications volontaires au lithium sous sa forme retard même après une semaine de la prise et l'insuffisance thérapeutique d'une seule séance d'hémodialyse.

## Abstract (en)

We report the case of a 47-years old patient, traited with lithium for manic-depressive psychosis over a period of twenty and admitted to hospital with a disorder of consciousness after suicide attempt with lithium overdose (ingestion of 30 tablets of Téralithe^®^ LP 400, delayed action galenic forms corresponding to 12 g of lithium carbonate), clinically improved after three hemodialysis sessions. This study illustrates the therapeutic role of hemodialysis in voluntary intoxications with extended release lithium even a week after the ingestion and the therapeutic insufficiency of a single hemodialysis session.

## Introduction

Depuis les années 1950 les sels de carbonate de lithium sont utilisés dans le traitement des maladies maniaco-dépressives [1]. Ils offrent une marge de sécurité thérapeutique acceptable dans le traitement des troubles de l'humeur sous réserve d'un contrôle régulier de la lithiémie plasmatique (intervalle thérapeutique entre 0,6 et 1,2 mmol/l). La forme « retard» apparue au milieu des années 90 permet une stabilité des taux de lithium circulant avec une seule prise journalière. Cet avantage pratique expose toutefois les patients à des effets prolongés en cas d'intoxication volontaire. Nous rapportons une observation d'intoxication volontaire par le Teralithe^®^ LP 400 (carbonate de lithium). Le traitement a nécessité le recours à l'hémodialyse. Y a-t-il un intérêt à dialyser un patient qui a présenté une intoxication volontaire au lithium après une semaine à fonction rénale normale et à diurèse conservée et à quel rythme?

## Patient et observation

Nous rapportons le cas d'un patient âgé de 47 ans, suivi depuis vingt ans pour une psychose maniaco-dépressive mis sous lithium. Son histoire remonte à 8 jours avant son admission au centre hospitalier universitaire Hassan II de Fès après une tentative de suicide au lithium. Il avait pris 30 comprimés de Téralithe^®^ LP 400, forme galénique retard correspondant à 12 g de carbonate de lithium. Il a présenté plusieurs épisodes de vomissements et des crises convulsives tonico-cloniques généralisées suite auxquels il a bénéficié d'un lavage gastrique et mis sous traitement symptomatique. Devant le non amélioration de son état clinique durant son hospitalisation qui a duré six jours, il fut référé au centre hospitalier universitaire Hassan II de Fès pour prise en charge. L'examen clinique à l'admission a objectivé un patient inconscient, score de Glasgow à 10, normotendu, en euvolémie clinique, diurèse conservée. L'examen neurologique est normal en dehors de l'altération de sa conscience. Le contrôle biologique réalisé montre une lithémie à 5 mmol/l, une fonction rénale normale sans troubles hydro-électrolytiques (créatinémie 10 mg/l, natrémie 135 mmol/l, kaliémie 3.5 mmol/l) et un bilan hépatique normal. Le patient a été transféré en réanimation; intubé ventilé sédaté sur des critères neurologiques et pulmonaires. La décision de mettre en œuvre une hémodialyse d’épuration est prise devant l'altération de sa conscience et la lithémie à 5mmol/l. Il a bénéficié de trois séances d'hémodialyse en cinq jours. Chaque séance a duré quatre heures par cathéter fémoral autorisant un débit sanguin moyen de 250 ml/min. L’état clinique du patient s'est nettement amélioré après les trois séances d'hémodialyse. Ainsi, la sédation a été arrêtée, et le patient extubé, avec un score de Glasgow à 15. La lithémie à la sortie du patient était à 0,5mmol/l.

## Discussion

Cette observation illustre la gravité de l'intoxication volontaire au lithium. Les manifestations cliniques en rapport avec une intoxication aiguë sont essentiellement neurologiques: elles vont de la somnolence, du tremblement léger, à la céphalée dans les formes minimes, à l'apathie avec difficultés d’élocution, tremblements musculaires, acouphènes dans les aspects modérés [2]. Dans les aspects les plus graves apparaissent coma, mouvements choréo-athétosiques, contractions musculaires diffuses, convulsions. S'associent également des troubles digestifs faits de nausées et vomissements ainsi que des douleurs abdominales comme cela a été le cas chez notre patient. Les manifestations cardiaques sont également rares, arythmie supraventriculaire, et plus souvent modifications électrocardiographiques isolées (QT long, modifications des ondes T). Il n'y a pas de manifestations rénales liées à l'intoxication aiguë par les sels de lithium, par contre il est fréquent d'observer au cours du traitement chronique un diabète insipide néphrogénique avec polyurie pouvant favoriser une déshydratation [2].

Les manifestations toxiques sont d'autant plus marquées qu'elles sont le fait de patients traités au long cours ingérant massivement les sels de lithium: la demi-vie plasmatique du lithium peut en effet passer de 12 heures à 58 heures suivant l'absence ou la présence d'un traitement chronique (plus d'un an) par le lithium [3]. Lors du traitement prolongé par le carbonate de lithium, l'accumulation intracellulaire du lithium d'une part ainsi que l'inhibition de la sortie du lithium intra-érythrocytaire d'autre part expliquent l'augmentation de la demi-vie du lithium plasmatique [4]. Ceci est indépendant de la forme galénique, qui, pour les formes à libération prolongée, retardent le pic d'absorption intestinale de 2 à 8 heures et l’élimination urinaire de 8 à 24 heures. D'autres facteurs peuvent intervenir dans l'accumulation du lithium: l'insuffisance rénale, les inhibiteurs de l'enzyme de conversion, les anti-inflammatoires non stéroïdiens et les diurétiques thiazidiques. Les critères thérapeutiques ont été définis sur les aspects cliniques et le taux de lithiémie [5, 6]: lavage digestif au polyéthylène glycol si le patient est admis précocement et sans troubles de la conscience, et réhydratation si nécessaire mais pas de bénéfice d'une diurèse forcée. La place de l'hémodialyse avec tampon bicarbonate qui est la technique d’épuration de référence [2, 3, 5], est définie: tous les patients traités au long cours par lithium si la lithiémie dépasse 4 mmol/l; si elle est entre 2,5 et 4 mmol/l, hémodialyse en cas de manifestations neurologiques sévères associées ou non d'instabilité hémodynamique ou neurologique. Si la lithiémie est inférieure à 2,5 mmol/l, la dialyse n'est indiquée qu'en cas d'insuffisance rénale chronique associée ou de délai de normalisation de la lithiémie supérieur à 36 heures [1, 5]. La clairance du lithium par l'hémodialyse est de l'ordre de 170 ml/min, elle n'est que de 20 à 40 ml/min par les reins propres; la dialyse péritonéale est très médiocrement active: clairance de 10 à 15 ml/min.

Notre observation objective le bénéfice de l'hémodialyse vu les manifestations neurologiques persistantes même après un délai d'une semaine après l'intoxication aiguë au lithium chez un patient qui a une fonction rénale normale et une diurèse conservée. La sécurité donnée par une seule hémodialyse précoce au cours des tentatives de suicide au lithium avec les formes retard est clairement insuffisante. Notre observation montre que les manifestations neurologiques ne régressent qu’à partir de la troisième séance. En outre, la lecture de la littérature confirme la gravité des tentatives de suicide chez les patients traités au long cours par le lithium. Les crises convulsives de ce patient ont été attribuées à l'intoxication par le lithium en l'absence d'antécédents, d'autres causes de convulsions, et devant la connaissance de cette complication au cours de tentative de suicide au lithium. Notre observation confirme que le délai d’élimination des sels de lithium dans sa forme retard peut prendre plus d'une semaine même si la fonction rénale est normale et la diurèse est conservée, d'où l'intérêt de dialyser les malades qui ont présenté une intoxication au lithium même après une semaine.

## Conclusion

Cette observation illustre l'intérêt thérapeutique de l'hémodialyse au cours des intoxications volontaires au lithium sous sa forme retard même après une semaine de la prise et l'insuffisance thérapeutique d'une seule séance d'hémodialyse. Cette information claire doit être donnée à tous ceux (réanimateurs, néphrologues ou autres) qui seront amenés à prendre en charge ces patients après ces tentatives de suicide.
